# SENSOR-BASED MONITORING OF PHYSICAL ACTIVITY IN OLDER ADULTS SIX MONTHS POST-FALL: MODERATING FACTORS

**DOI:** 10.1093/geroni/igae098.4073

**Published:** 2024-12-31

**Authors:** Laura Schmidt, Tim Stuckenschneider, Robert Kwiecien, Tania Zieschang

**Affiliations:** Carl von Ossietzky University Oldenburg, Oldenburg, Niedersachsen, Germany; Carl von Ossietzky University Oldenburg, Oldenburg, Niedersachsen, Germany; University of Muenster, Muenster, Nordrhein-Westfalen, Germany; Carl von Ossietzky University Oldenburg, Oldenburg, Niedersachsen, Germany

## Abstract

Older individuals who have experienced a fall often experience concerns about falling, impaired activities of daily living, and reduced physical activity (PA). Changes in PA following a fall are often influenced by moderators such as age or fall history; however, these moderators remain understudied. Therefore, this project investigates the association between moderators and changes in PA in older adults after a fall. Participants aged ≥60 years were recruited if they presented to the emergency department following a fall without hospital admission. Moderators were collected through a home-based geriatric assessment conducted within four weeks after the fall. PA was measured within four weeks and six months after the fall using an accelerometer (activPAL4, PAL Technologies Ltd., UK). Associations between moderators and changes in PA were calculated fitting Linear Mixed Model (LMM) in R (R Core Team, 2023 Version 4.3.2 (2023-10-31). A total of 178 valid datasets were analyzed. LMM analysis revealed a significant average increase in step count over time (p=0.006). Notably, female participants showed a significant larger increase in steps per day (+1,777.9, 95% CI: 292.2 – 3263.7, p=0.019) than male participants. In women, fall history (95% CI: -1009.5 – (-207.4), p=0.003) and age (95% CI: -315.8 – (-82.5), p< 0.01) were negatively associated with step counts. Given the healthcare challenges associated with falls in older adults, our findings underscore the importance of early identification of moderators in emergency department settings. Identifying these factors may enable targeted interventions for individuals at risk of functional decline post-fall, potentially preventing further functional decline.

